# The Use of Composite Flaps in the Management of Large Full-Thickness Defects of the Lower Eyelid

**DOI:** 10.1097/MD.0000000000002505

**Published:** 2016-01-15

**Authors:** Shuo Fang, Chao Yang, Yuntong Zhang, Chunyu Xue, Hongda Bi, Haiying Dai, Xin Xing

**Affiliations:** From the Department of Plastic and Reconstruction, Changhai Hospital Affiliated to the Second Military Medical University, Shanghai, PR China (SF, CY, CX, HB, HD, XX); and Department of Emergency and Trauma, Changhai Hospital Affiliated to the Second Military Medical University, Shanghai, PR China (YZ).

## Abstract

To describe a modified surgical procedure that uses a combination of the tarsoconjunctival flap, orbicularis myocutaneous advancement flap, and paranasal-island flap to correct extensive full-thickness lower eyelid defects in functioning eyes.

From May 2010 to December 2013, a total of 15 patients had reconstructive surgeries of large to giant lower eyelid defect, with an average 19-month follow-up. The musculocutaneous flaps were harvested from both orbicularis and paranasal regions and clinical outcomes were recorded and analyzed.

No major complications were observed in any of the patients. All the patients showed aesthetic eyelid contour, good color, and texture match as well as no obvious scar formation. The mean Marginal Reflex Distance-2 measured 4 months after surgery was 4.9 ± 0.4 mm.

Reconstruction of a large defect in the lower eyelid with a tarsoconjunctival flap and the composite neighboring musculocutaneous flaps is a reliable and reproducible method. With proper design and well-executed precision, excellent functional and aesthetic results can be achieved by this elegant procedure without any major complications.

## INTRODUCTION

There are various causes for eyelid and/or canthus defects.^1^ Basal cell carcinomas, for instance, account for approximately 90% of eyelid tumors and most frequently arise on the lower eyelid and the medial canthus, and least often near the lateral canthus.^2^ The basic rule in tumor surgery of the eyelids is to ensure radical excision, even when such treatment involves the sacrifice of several components of the eyelid and neighboring structures because inadequate excision could lead to a high rate of recurrence.^3,4^ Concomitantly, full-thickness lower palpebral reconstruction can be complex and challenging. Eyelids are composite structures, formed by an anterior and a posterior lamella, with the anterior lamella comprising the skin and the orbicularis and the posterior lamella comprising the tarsus and the conjunctiva. Reconstruction of eyelid defects requires the replacement of the internal mucosa layer and the external musculocutaneous layer covered with skin. These structures must be repaired, if possible with matching tissues with respect to composition, size, color, and pliability, leaving minimal donor site morbidity and inconspicuous scars.^1,5,6^

It is possible to find in the literature various methods to reconstruct full-thickness defects of the lower eyelid due to the removal of infiltrating tumors. If the defect is small and the skin lax, primary closure with undermining of the adjacent skin and orbicularis is ideal. In patients with defects involving less than 33% of the lower eyelid, a number of techniques, including the Tenzel rotation flap, the sandwich technique, and the adjacent tarsoconjunctival flap combined with skin graft, have been reported.^1,7–9^ However, most of these are performed as a one-step procedure and are not applied for reconstruction of vertical defects larger than 10 mm, which may lead to a high rate of postoperative lid retraction.^1,3,8,10–16^ For extensive defects, which involve greater than 75% of the lower eyelid, the number of ideal techniques restoring both function and appearance of the eyelid is limited.

In this study, we describe a modified technique, using the upper tarsoconjunctival advancement flap combined with the composite neighboring musculocutaneous flaps, for a large to giant lower eyelid reconstruction. A detailed description of the procedure and clinical outcomes are reported and evaluated in this paper.

## METHODS

The study protocol was approved by the local ethics review committee of the Second Military Medical University, and all participants provided us with written informed consent prior to inclusion.

From May 2010 to December 2013, a total of 15 patients had reconstructive and functional restoration surgeries of lower eyelid defects, with follow-up ranging from 12 to 26 months. Patients underwent uncomplicated surgical reconstruction performed by 1 senior surgeon (XX). Six patients were women and 9 were men, and their age ranged from 32 to 79 years (average, 60 y). All 15 patients had undergone surgical resection of a malignant tumor. None of the patients had evidence of regional or distant metastasis. All tumors were excised with clear margins and were diagnosed as basal cell carcinomas using frozen section controls. Patients had large to giant (greater than 70%) unilateral full-thickness lower eyelid defects and could not completely close their eyelids. The horizontal defect size ranged from 2.8 to 4.0 cm, and the vertical defect size ranged from 1.3 to 2.3 cm. The defects in 5 patients involved the medial canthus as well as the adjacent medial lower eyelid. Eight involved the temporal aspects of the lower eyelid. Patient demographic information, including sex, age, defect size, and other characteristics, is summarized in Table 1.

**TABLE 1:**
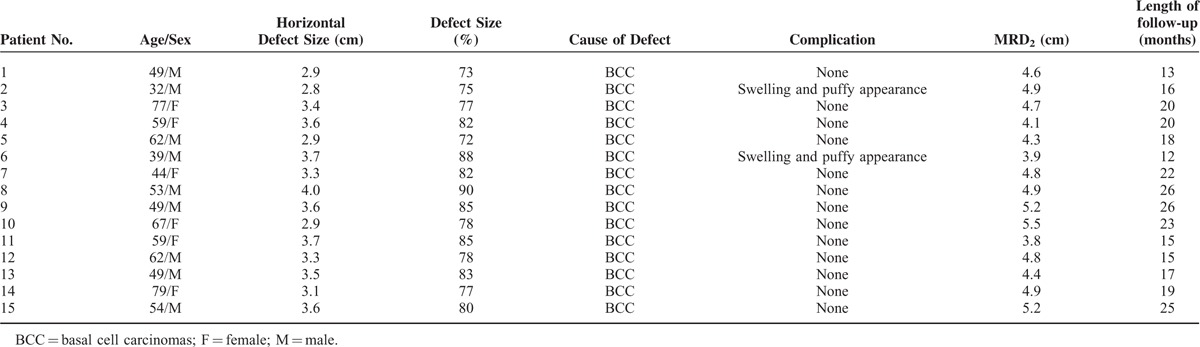
Patients’ Characteristics, Complications, and Length of Follow-Up

### Surgical Technique

All patients were operated on under general anesthesia. The planned surgical excision was then marked. Carcinomas were excised completely with adequate margins according to oncological principles concerning full-thickness specimens and were examined by frozen section examination immediately. Reconstruction proceeded after confirmation of tumor-free margin. The defects ranged from 70% to 90% of the lower eyelid width. The tumor was removed with a radical intent, while functional planning of the reconstructive project was considered concurrently.

A no. 15 Bard-Parker blade was used to incise a tarsal conjunctival flap beginning 3 mm superior to the upper eyelid margin and covering the entire horizontal length of the upper tarsus. An ipsilateral tarsoconjunctival flap with the length of 19 to 22 mm comprised of tarsal plate and conjunctiva was undermined with sharp scissors to the superior fornix. The medial and lateral aspects of the flap were cut to mobilize the flap, which was then advanced to the lower eyelid defect site. The inferior cut edge of the tarsus in the flap was sewn to the inferior edge of the remaining posterior lamella with a running 6-0 absorbable braided polyglactin suture.

Two composite flaps were placed arbitrarily according to the need for proper construction of the anterior lamella and margin contour. An orbicularis myocutaneous advancement flap was designed along the direction of fishtail wrinkles beside the temporal portion of the eyelid defect. The borders of the flap were incised to the submuscular plane, and the muscle was split in the direction of the muscle fibers to spare the function of the remaining muscle. Care was taken to avoid damaging the pedicle. The flap was elevated and transposed to remove tension on the defect. A paranasal island pedicel flap was designed on the medial side of the defect with a similar procedure as that previously described. The paranasal perforators of the angular artery should be identified and protected carefully to ensure the blood supply of submuscular pedicle. The lines of incision and suture, especially for the conjunction of the composite flaps at the mid-lower eyelid, were shaped into a Z-fashion and conducted along the Langer lines to reduce tension and to improve the aesthetic result. The donor sites of both flaps were sutured directly in 2 layers without tension. Drain was positioned under the flaps and removed after about 3 days.

The hospitalization days differed among patients, based on each patient's individual conditions. Antibiotics and corticosteroid were administered for 2 to 3 days after the surgery. Stage 2 was performed 5 to 7 weeks later. Sharp Westcott scissors were used to sever the tarsoconjunctival flap, and the redundant flap was excised. Follow-up examinations were performed 1, 3, 6, and 12 months after surgery and then annually thereafter or more frequently. Changes in functional and aesthetic outcome, as well as any discomforts or complications, were recorded.

**FIGURE 1:**
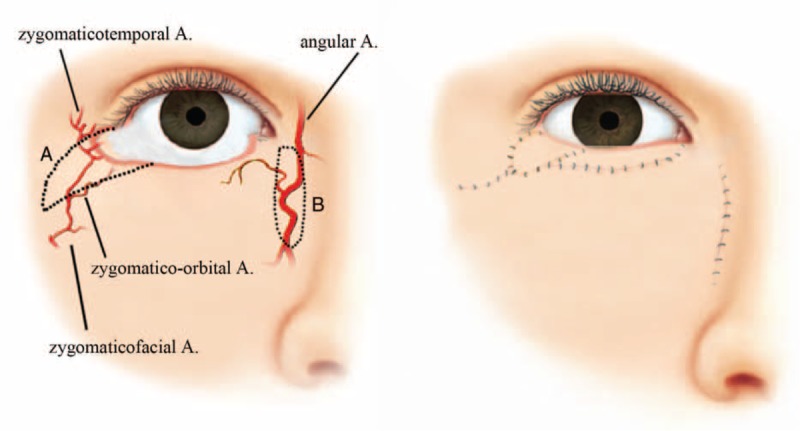
An illustration showing the surrounding arterial anatomy and the design of the orbicularis myocutaneous advancement flap and paranasal island flap for the anterior lamellar defect (left). The final postoperative appearance (right).

**FIGURE 2:**
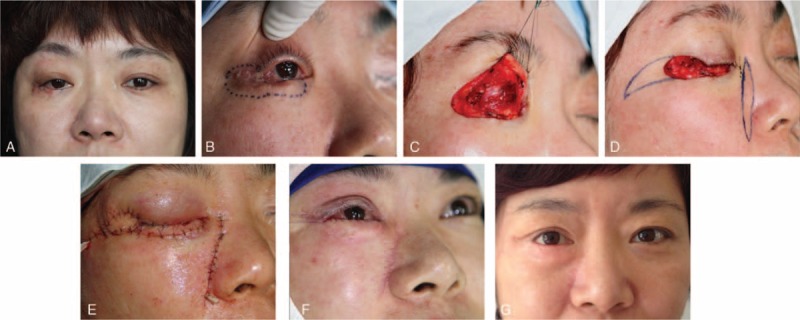
A, Preoperative clinical photograph showing a 44-year-old woman suffering from basal cell carcinomas involving the right lower eyelid. B, Resection creating a full-thickness defect involving 82 percent of the lower eyelid and lateral canthus. C, Posterior lamellar defect was corrected by tarsoconjunctival flap. D, Design of the composite flaps (2 cm × 1.5 cm orbicularis myocutaneous advancement flap and 2 cm × 3.5 cm paranasal island flap) and preoperative marking for the anterior lamellar defect. E, Preoperative view of the patient. F, Secondary division of the tarsoconjunctival flap pedicle was performed 5 weeks later. G, Postoperative view after 22 months showing aesthetic eyelid contour with no obvious scar formation in both donor sites and reception sites.

Illustration and representative images of the surgical procedure are shown in Figures 1 and 2.

## RESULTS

In this study, 15 patients who had unilateral large full-thickness lower eyelid defects due to surgical resections of malignant tumors were the recipients of reconstructive surgery.

All tumors were removed with safe margins, and no relapse has been recorded to date. The mean follow-up time was 19 months (ranging from 12 to 26 months). In each postoperative follow-up, patients were evaluated in terms of eyelid function, color, and texture. All patients were satisfied with the outcomes, and their defects were well reconstructed both aesthetically and functionally. No major complications, including partial or total flap necrosis, signs of infection, venous congestion, and hematoma were observed in any of the patients. Ectropion, entropion, postoperative irritation, or ulceration of the conjunctiva and cornea were not observed. Prolonged lower lid swelling and a slight puffy appearance were noted in 3 patients. The swelling and puffy appearance resolved completely 2 months after surgery. The mean MRD_2_ measured 4 months after surgery was 4.7 ± 0.4 mm. All patients showed aesthetic eyelid contour, good color, and texture match and were able to open or close their eyes freely with no obvious scar formation. Donor sites were all healed primarily, and scars were not obvious.

## DISCUSSION

Basal cell carcinoma is the most common cutaneous malignancy of periocular region.^2,17^ The treatment of basal cell carcinoma as well as trauma result in eyelid defect inevitably.[Bibr R10] The full-thickness reconstruction of the eyelid demands the restoration of 2 distinct layers that have different anatomy and function. For reconstructed tissues to survive, it is essential that at least 1 lamella have an intact blood supply.^1,6,8^

Considering the normal structure of lower eyelid, reconstruction of anterior and posterior lamella by harvesting tissue from various sites has been established. The most satisfactory tissues for inner layer reconstruction are autogenous tarsus and attached conjunctiva, which have the anatomical features of the normal eyelid.^13^ Some defects can be closed only with skin sutures, but deeper defects and those under tension require layered closure.^10,11,16,19,20^ The use of a 1-step procedure such as the free tarsoconjunctival graft and flap would inevitably cause eyelid retraction.^21,22^ The Mustarde flap as well as the modified rotational advancement cheek flap can wrap a large defect consisting of as much as 75% of eyelid by providing a more physiological structure and little disturbance of the donor site. However, drawbacks including retraction, extensive tissue dissection, and facial nerve damage have been reported.^23–25^

Taking both therapeutic and aesthetic effects into consideration, the authors present a modified surgical procedure that uses a combination of tarsoconjunctival flap, orbicularis myocutaneous advancement flap, and paranasal island flap to correct extensive full-thickness lower eyelid defects. The tarsoconjunctival flap, described by Hughes in 1937, is one of the favorable options for repair of the posterior lamella of the lower eyelid. Although it is a 2-stage procedure, the tarsoconjunctival flap can be of special use in larger defects because it offers conjunctiva as well as a tarsal pedicle, which connects the upper and lower eyelids for weeks.^9,26^ Multiple modifications have been described to eliminate the risk of lower eyelid ectropion, eyelid margin entropion, and upper eyelid retraction. Although a number of different techniques, such as local or regional flaps, free flaps, and skin graft, have been utilized to restore the superior eyelid in recent studies, outcomes following these techniques have varied.^9,15,21,23,26^ Full-thickness skin grafts of upper eyelid skin, postauricular or supraclavicular skin, for example, may be utilized if the mucosa layer has an abundant vascular supply. However, it does not give quite as good of a color and thickness match as a flap. For a better aesthetic outcome, local myocutaneous pedicle flaps are preferred because they tend to be readily available and thin, and they provide a superior color match. Furthermore, it is important to individualize the design of the flap/graft, according to defect localization, size, depth, and other patient characteristics. In the cases presented here, patients suffered from quite extensive defects of the lower eyelid and the lateral/medial canthus region. Nine patients had full-thickness defects of the lower eyelid of greater than 75%, and 12 cases involved the lateral or medial canthus. Patients had high expectations concerning the aesthetic outcome. Single or distant flaps are not only potentially limited in size especially in the horizontal direction, but have a high risk of morbidity in both donor and recipient sites as well. In our technique, the combination of orbicularis myocutaneous advancement flap, and paranasal island flap is utilized to repair the anterior lamella. In comparison with other methods, the use of 2 composite local flaps in the anterior layer, instead of 1, allows for several benefits. First, the most important advantage of this composite flap is that it is usable in nearly all defects of the lower eyelid, especially for those with a full-thickness defect greater than 75%. The skin and subcutaneous tissue at both donor sides are loose, sufficient in size and easy to design without horizontal tension. Thus, this technique combines the benefits of repairing lower periorbital defects of considerable length and height while maintaining a superior lid margin architecture As a result, the incidence of postoperative entropion and ectropion, common in other procedures, was not observed in this study. Second, the anterior layer deficit is replaced with a nearby local flap, which is a highly preferred option in skin reconstruction due to its similarity in color, texture, and thickness. With experimental planning, the incision and saturation can not only be designed along the direction of the Langer skin lines as far as possible but also be hidden in the nasolabial fold and fishtail lines according to the shape of defect, leaving almost invisible donor-site scars. The optical symmetry can also be considered in advance for improved aesthetic outcomes. Third, the composite local flaps are advantageous in providing a rich blood supply to the bilateral orbital region, which means improvements in the chance and rate of healing compared to grafts, with a reduced risk of ischemic necrosis and chromatosis. Finally, compared with other large single or distant flap methods, although more incisions required, the operation time is equivalent and the approach is feasible and easy to perform.

Despite all these advantages, several recommendations when using the composite flaps should be heeded: the horizontal tarsal incision should parallel the edge of the eyelid and should be kept at least 4 mm away from the upper eyelid; the tarsoconjunctival flap should consist only of tarsus and conjunctiva, with levator palpebrae superioris and Mueller muscle intact in the upper eyelid; the lacrimal system including the lacrimal gland and its secretory ductules should be taken care of while excising and reconstructing the medial canthal lid; and the lateral pedicle of the flap should be widened enough to ensure sufficient blood supply.

The disadvantages of this technique include the absence of eyelashes and the preclusion of the 2-stage procedure, which requires the occlusion of the visual axis for 6 weeks and a secondary operation to create a conjunctiva-lined lid margin. Although the anterior pedicle flaps have abundant vascular supply, free tarsoconjunctival flaps or cartilage grafts are not recommended due to the high risk of flap failure and retraction.

## CONCLUSION

The reconstruction of a large defect in the lower eyelid with a tarsoconjunctival flap and composite neighboring musculocutaneous flaps is a reliable and reproducible method, which has been rarely reported in the literature. It also has the advantages of good color and thickness match, rich blood supply, and restoration of a near-normal lid margin. With proper design and well-executed precision, excellent functional and aesthetic results can be achieved by this elegant procedure without any major complications.
